# Noise-induced hearing loss and hearing protection: Attitudes at a South African coal mine

**DOI:** 10.4102/sajcd.v71i1.966

**Published:** 2024-01-17

**Authors:** Kavitha Naicker

**Affiliations:** 1 School of Health Systems and Public Health, Faculty of Public Health, University of Pretoria, Pretoria, South Africa

**Keywords:** noise, noise-induced hearing loss, hearing protection devices, attitudes, beliefs

## Abstract

**Background:**

Negative attitudes and beliefs are major contributing factors to the rising numbers of noise-induced hearing loss (NIHL) cases in coal mines both locally and internationally. International literature confirms limited knowledge surrounding employees’ attitudes and beliefs regarding NIHL and hearing protection devices (HPDs), hence the need for the study.

**Objectives:**

To ascertain the attitudes and beliefs about NIHL and HPD use among employees at a large scale underground coal mine in Mpumalanga.

**Method:**

A descriptive and exploratory cross-sectional study was conducted using a self-administered questionnaire, developed by the National Institute for Occupational Safety and Health (NIOSH) on Beliefs about Hearing Protection and Hearing Loss (BHPHL). Participants (*n* = 241) included employees from a coal mine in Mpumalanga, South Africa.

**Results:**

Out of 241 completed surveys, this study found that 84% were aware of when to replace earmuffs; 95% believed wearing HPDs could prevent hearing loss in noisy environments; 83% felt their hearing was impacted by loud noise. Additionally, 86% mentioned discomfort from earmuff pressure; 95% emphasised HPD importance; and 95% used HPDs around loud sounds. Moreover, 98% knew how to properly wear earplugs, while lower education levels were linked to higher susceptibility to NIHL.

**Conclusion:**

The study identified positive attitudes towards NIHL and HPD use, but existing NIHL cases must be acknowledged. Organisations can use the findings to develop tailored hearing conservation programmes (HCP), including education, involving employees in protection decisions and promoting diligent HPD usage.

**Contribution:**

This study contributes to the limited literature on noise perceptions, NIHL, and HPD use in mining, emphasising the impact attitude has on HPD use and assessing the effect of miners NIHL knowledge on compliance. The findings, unique to coal mining, hold significance for enhancing hearing conservation and reducing NIHL.

## Introduction

Noise is an unwanted sound that is frequently of increased volume, which is unpleasant and undesirable and can be defined by its intensity and frequency (Nair, 2014). As a result of its painless nature, if left undetected and untreated, prolonged exposures to noise result in a permanent, progressive and degenerative process, which can lead to noise-induced hearing loss (NIHL) (Nair, 2014). In South Africa, around 73.2% of mineworkers are exposed to noise levels in excess of the legislative value of 85 dBA (Department of Employment and Labour [DEL], 2020). Similarly, in the United States, 80% of mineworkers are exposed to noise levels in excess of the legislative record of 85 dBA (Masterson & Themann, 2018), while 22 million workers around the world are exposed to excessive amounts of noise (Sun et al., 2019). Sun and colleagues (2019) further add that 18% of these 22 million noise-exposed employees go on to develop NIHL. The given data suggest that the problem of preventing NIHL is a global problem that is not restricted to a particular area or business.

In certain high-noise-generating industries such as mining and quarrying, manufacturing, construction and farming, NIHL is very common and is reported to be one of the top three occupational diseases faced among South African miners (DEL, 2020). The National Institute for Occupational Safety and Health (NIOSH) has identified NIHL as a research priority because of its significant impact as one of the top 10 occupationally acquired diseases internationally (NIOSH, 2019). In South Africa alone, the increasing number of NIHL cases in mines reported by the Department of Mineral Resources and Energy in 2022 is evidenced by the 5.2% rise in NIHL cases, with the number increasing from 738 in 2020 to 776 in 2021 (Department of Mineral Resources and Energy [DMRE], 2022). Moreover, the inability to hear sounds accurately as a consequence of NIHL has the potential to cause increased accidents and injuries at work, home and personal settings (Chen et al., 2020). This is detrimental to the worker and everyone around them, threatening their safety and wellbeing (Chen et al., 2020). Thus, NIHL not only affects one’s ability to hear in a physical capacity but also has profound effects on the person mentally, emotionally, socially, psychosocially and psychologically (World Health Organization [WHO], 2019).

Currently, a dearth of information and published literature explains the disease process, in addition to preventative measures in specific high-noise-risk industries. However, very few studies have focused on the attitudes of employees towards the disease and the use of hearing protection devices (HPDs). Moreover, previous studies have primarily focused on the attitudes and beliefs of young individuals towards noise and hearing loss (HL) such as those conducted by Keppler et al. (2015); Keppler et al. (2015) and Gilles et al. (2013). However, there is a lack of literature addressing the attitudes and beliefs of the working population and these aspects are often overlooked. The existing limited studies indicate that individuals generally assess the severity of the consequences of NIHL as more negative on average (Hansia & Dickinson, 2010; Mizan et al., 2014; WHO, 2020). This suggests that they are aware of the diminished communication skills associated with NIHL. However, these studies also highlight that being aware of the consequences of NIHL does not necessarily lead to behavioural change. Therefore, it is important to understand employees’ attitudes and beliefs regarding NIHL and the use of HPDs in order to effectively develop programmes and strategies. By gaining insights into these attitudes and beliefs, employers can address underlying issues and contribute to a better understanding of the problem. This understanding can lead to the development of effective measures to control occupational noise exposure and reduce the incidence of NIHL in South Africa and globally. Therefore, the researcher conducted this study to explore the attitudes and beliefs of coal mine workers towards NIHL and the use of HPDs, using the Beliefs about Hearing Protection and Hearing Loss (BAHPHL) questionnaire developed by NIOSH. The questionnaire explored various factors such as susceptibility to HL, the severity of its consequences, the benefits and barriers to preventive actions, behavioural intentions, social norms and self-efficacy (Appendix 1).

The findings from this study can contribute to the development of comprehensive Hearing Conservation Programs (HCPs) that align with the best practices for preventing NIHL. In addition, the research outcomes can inform the improvement of training and education programmes in industries. The data collected will also contribute to the existing global and local literature, influencing the formulation of policies, procedures and potentially legislation related to hearing conservation. These efforts aim to support the South African Mine Health and Safety Council’s (MHSC) commitment to enhancing HCPs by 2024.

## Research methods and design

### Study design

This study followed a quantitative, descriptive and exploratory approach, which was cross sectional in nature, with a questionnaire used as a data collection tool. Quantitative research was chosen, as its numerical and statistical nature allowed for an enhanced perspective that generated effective conclusions and inferences from a representative sample to explain and analyse data that were collected (Brink et al., 2018).

### Study setting

The research setting was one of six large-scale coal mines (*n* = 6) owned by a specific mining company. These mines supply feedstock to various other local and international industries such as that of chemical companies and those companies that generate petrol, electricity and steam (Sasol, 2019).

The researcher selected coal mining as a research setting, as research indicates that coal mining exhibits a greater proportion of miners with hearing impairment compared with other types of mining (Roberts et al., 2017; Sun & Azman, 2018; Sun et al., 2019). Furthermore, coal mines were more prone to receiving citations for violating noise restrictions when compared with miners in other sectors (Sun & Azman, 2018).

While the study did not include the participation of the five additional mines, it is important to note that these mines did not exhibit any significant differences in terms of noise levels and company policies and procedures compared to the mine that was included in the study as these mines form part of one organisation. This crucial detail ensures that the sample used in the study remains representative of the mines excluded, as the excluded mines can be considered to have similar characteristics to the one that was studied.

### Pilot study

To ensure the validity of the questionnaire, a pilot study was carried out using a small sample of 20 randomly selected participants from the same study area who willingly agreed to take part. This pilot study aimed to assess the questionnaire’s clarity, readability, feasibility and suitability for the research. The feedback obtained from the pilot study, particularly regarding the difficulty, length and format of the questionnaire, was taken into consideration during the development of the final version.

The pilot study revealed that the questionnaire was well understood, as all questions were filled in completely and no questions or queries were raised thereafter. Therefore, there was no need for any amendments in the questionnaire. However, the author did opt to add emoticons to the Likert scale. Emoticons were chosen because studies have shown that they improve emotional expression and comprehension while also triggering emotional responses (Evans, 2015; Phan et al., 2019; Tauch & Kanjo, 2016). By substituting a visual representation or icon that captures the same emotional tone as the text label, serving as a non-verbal indicator, for conventional lexical indicators such as ‘like’, ‘dislike’ and ‘unsure’, Phan et al. (2019) suggested that interest can be assessed. Furthermore, Tauch and Kanjo (2016) found that these non-verbal cues are important because measurement techniques incorporating them more accurately capture the emotional dimensions of attitudes, such as satisfaction. Therefore, the author saw it fit to use emoticons to enhance comprehension and affect of the questionnaire. The results of the pilot study were not included in the main study of 241 participants.

### Study population and sampling

In this study, the population referred to all the employees working in the selected mine and the sample included all employees who met the inclusion criteria and agreed to participate in the study. The inclusion criteria included all employees who could read and write English, were full-time employees as well as being manual laboured employees, for example, miners, operators, safety officers, managers, and so on. All administrative and office personnel, such as administrators and HR personnel, were excluded from the study as they do not go underground, neither are they exposed to excessive noise on surface.

### Sampling method

The sampling procedure followed a convenience sampling technique. Convenience sampling was chosen as it has been shown to be a quick way of collecting data, in addition to being easy and inexpensive whereby participants are readily available (Brink et al., 2018).

This study was conducted at the weekly Production and Services communication meeting. This meeting entailed a mass gathering of all categories of employees (excluding administrative surface workers). This site was selected for data collection as it ensured that all employees had an equal chance of participating in the study. All employees present at the communication session met the inclusion criteria. This method of drawing participants ensured that every employee had an equal chance at participation, thereby ensuring representativeness of the data collected.

The BAHPHL questionnaire was thoroughly explained to the participants by the researcher, and any queries or concerns about the questions were addressed. This approach aimed to familiarise the employees with the questionnaire, resulting in a reduced time to complete it. Following this, the questionnaires were distributed to the employees for completion. Employees were made aware of the voluntary nature of filling in the questionnaire.

The completion of the questionnaire typically took around 10–15 min. To ensure clarity and assist participants, the researcher was present on-site during data collection to address any questions or confusion related to the questionnaire. Before participants dropped their completed questionnaires into the drop box, the researcher quickly reviewed each questionnaire to ensure completeness and to avoid any data loss. If any questions were left unanswered, the participant was asked to provide responses for those specific questions. Questionnaires were filled in up until the sample number had been reached.

### Sample size

Using the Raosoft computer package, allowing for a 5% marginal error, with a 95% confidence interval from a population size of 1500, a sample size of 306 was calculated. However, this sample size of 306 was not met as employees became reluctant to fill in the questionnaire stating that it took time, and they were pressurised to go underground because of production needs. And therefore, the researcher was left with 241 completed questionnaires, which equated to a response rate of 79%. Vianna (2021) states that an adequate response rate for in-person surveys should be at least 57%. Furthermore, in consultation with the biostatistician, it was agreed that the obtained questionnaires were sufficient for the study.

### Data collection tool

In order to determine the attitudes and beliefs of employees towards NIHL and the use of HPDs, this study made use of the original English version of the BAHPHL scale developed by NIOSH. This questionnaire assesses the attitudes, beliefs and behavioural intentions of workers pertaining to the prevention of NIHL, which encompassed the research objectives for this study. Moreover, this questionnaire was selected as it was a good fit for the study aim. Its subscales include that of the perceived susceptibility to HL; the perceived severity of consequences of HL; the perceived benefits of preventative action; the perceived barriers to preventative actions; behavioural intentions; social norms and self-efficacy. These subscales were measured on a five-point Likert-type scale, whereby: ‘1’ implied a response of Totally Agree, ‘2’ meant Agree, ‘3’ meant Unsure, ‘4’ meant Disagree and finally ‘5’ meant Totally Disagree. Responses ‘1’ and ‘2’ were grouped into an overall ‘agree’ response, while responses ‘4’ and ‘5’ were grouped into an overall ‘disagree’ response. Moreover, responses in the lower ranges (i.e., 1 and 2) implied a negative attitude, whereas responses in the higher ranges (i.e., 4 and 5) implied a positive attitude towards NIHL and HPDs.

This questionnaire has previously been used and validated in South Africa (Keppler et al., 2015) and internationally such as in New Zealand (Reddya et al., 2021), Colorado (Hickey, 2019) and Sweden (Svensson et al., 2004). In addition to the questionnaire’s internal consistency being previously investigated by Gilles et al. (2013) and Keppler (2010), the results of a research investigation into the test-retest reliability of the BHPHL questionnaire revealed that there was no noteworthy association between the test and retest, and the variances in the scores of the BAHPHL questionnaire (as determined by Pearson correlation, *p* > 0.05) (Sofie et al., 2018). Additionally, a paired *t*-test conducted on the complete BAHPHL questionnaire and its subscales indicated no significant disparities in average scores between the initial test and the subsequent retest (*p* > 0.05) (Sofie et al., 2018).

### Data analysis

Data were firstly entered onto an Excel spreadsheet based on the responses received from the BAHPHL questionnaires before being imported into Stata 15 intercooled edition. The data analysis process involved several aspects. Firstly, the demographic characteristics of the participants were examined using the Shapiro-Wilk test for normality to assess the distribution of responses. Descriptive statistics were then employed to summarise the data.

Secondly, the responses received for each of the seven subscales were analysed. The 31 questions were grouped under their respective subscales, including susceptibility to HL, severity of consequences, benefits of preventative action, barriers to prevention, behavioural intentions, social norms and self-efficacy. Responses were tallied using a five-point Likert scale.

Thirdly, it was determined whether the overall responses for each subscale were positive or negative. A negative overall response was calculated by combining all scores in the Disagree and Totally Disagree categories of the Likert scale (4 and 5) and dividing them by the total number of responses. A positive response was obtained by combining all the responses in the Totally Agree and Agree categories (1 and 2). The subscale with a higher value of positive responses was considered to have an overall positive response to that subscale. Descriptive statistics were also used to describe the data.

Lastly, the data were checked for normality, and multivariate logistic regression was conducted to explore associations between demographic variables (such as age, gender, education, job duration and job description) and the seven subscales. Descriptive data from tables were utilised to interpret these data.

### Ethical considerations

Ethical clearance to conduct this study was obtained from the University of Pretoria Faculty of Health Sciences Research Ethics Committee (No. 822/2019). Organisational approval was received from the company in which the study took place, as well as approved consent from each employee that participated in the study.

## Results

There was a 79% response rate, wherein all 241 questionnaires were included in the analysis. Table 1 shows that most participants were in the age group of 30–39 years (45%), of which males comprised the majority of of the sample (83%). Just under half (48%) of the total sample were married, while just over half (51%) reported to have a highest qualification of Grade 9–12. Most respondents worked for the company for 1–4 years and 5–9 years (31.5% for each category). The majority of participants (68%) in this study were of an operator job category.

**TABLE 1:** Demographic characteristics of participants.

Variable	*n*	Population (%)
**Age (years)**		
20–29	44	18.0
30–39	108	45.0
40–49	48	20.0
50–59	38	16.0
> 60	3	1.0
**Gender**		
Female	41	17.0
Male	200	83.0
**Marital status**		
Never married	58	24.0
Married	116	48.0
Divorced	6	3.0
Widowed	0	0.0
Living with partner	61	25.0
**Highest level of education**		
Grade 5–8	38	16.0
Grade 9–12	124	51.0
Certificate (post high school)	40	16.0
Diploma	28	12.0
Degree	9	4.0
Masters	2	1.0
**How long have you been working for the company**		
< 1 year	19	8.0
1–4 years	76	31.5
5–9 years	76	31.5
10–14 years	34	14.0
> 15 years	36	15.0
**Which best describes your job**		
Operator	164	68.0
Artisan	12	5.0
Electromechanic	12	5.0
Miner	23	10.0
Engineer	5	2.0
Manager	25	10.0

The findings of the study indicate that majority of participants displayed positive attitudes and beliefs towards various aspects related to HL prevention. In terms of susceptibility to HL, 86% agreed that their co-workers wear HPDs when working in loud noise, and a significant number (63%) knew when to replace their earplugs (Table 2). Additionally, 96% agreed that they could protect their hearing by wearing HPDs. Overall, 55% of participants had a positive response towards susceptibility to HL.

**TABLE 2:** Summarised results of the Beliefs about Hearing Protection and Hearing Loss questionnaire.

Question	Agree (%)	Unsure (%)	Disagree (%)
Most of my co-workers wear hearing protectors when they work around loud noise.	86	8	6
I’m not sure how to tell when earplugs need to be replaced.	30	7	53
If I wear hearing protection, I can protect my hearing.	96	2	2
I am convinced I can prevent hearing loss by wearing hearing protectors whenever I work in loud noise.	95	3	2
I believe that daily exposure to loud machinery and tools will eventually damage my hearing.	92	3	5
I think it would be a big problem if I lost my hearing.	95	3	2
I know when I should use hearing protectors.	98	1	1
I think my hearing is being hurt by exposure to loud noise at work.	73	5	12
I plan to wear hearing protection when I work near loud noises.	98	0	2
I think earmuffs put too much pressure on my ears.	86	8	6
I think wearing hearing protectors every time I am working in loud noise is important.	97	1	2
Most of my co-workers think it is a good idea to wear hearing protectors in hazardous noise.	91	4	5
If co-workers asked me, I would be able to help them wear hearing protectors correctly.	97	2	1
I wear hearing protectors whenever I work around loud noise.	95	2	3
I don’t think it would be such a big handicap to lose part of my hearing.	34	3	63
I believe I know how to fit and wear earplugs.	98	1	1
Hearing protectors are uncomfortable to wear.	28	4	68
Losing my hearing would make it hard for people to talk to me.	92	3	5

Regarding the severity of the consequences of HL, a high percentage (95%) agreed that they could prevent HL by wearing HPDs in noisy environments. Similarly, 92% acknowledged that daily exposure to loud machinery and tools could lead to hearing damage, while 95% agreed that HL would be a significant problem (Table 2). The majority (67%) had a positive response towards the severity of the consequences of HL.

In terms of the benefits of preventive action, a significant proportion (98%) agreed that they knew when to wear HPDs, and 73% believed that their hearing was being harmed by exposure to loud noises at work (Table 2). Furthermore, 98 % expressed the intention to wear HPDs in noisy conditions. Overall, 78% showed a positive response towards the benefits of preventative action.

The study also examined barriers to preventive action, revealing that 86% agreed that earmuffs put too much pressure on their ears, with 59% having positive responses to barriers to preventative action.

Regarding behavioural intentions, a high percentage (97%) agreed that wearing HPDs whenever working in loud noise is important, and 91% believed that their co-workers also valued wearing HPDs in hazardous noise environments (Table 2). In addition, 97% expressed the intention to assist their co-workers in correctly wearing HPDs. Overall, 63% of participants displayed positive responses towards behavioural intentions.

Social norms surrounding HPD use were also explored, with 95% agreeing that they wear HPDs when working in loud noise and 63% disagreeing with the idea that losing part of their hearing would not be a big problem (Table 2). In general, participants had a positive response (64%) towards social norms.

Regarding self-efficacy, 98% of participants expressed confidence in their ability to fit and wear earplugs correctly. While 68% disagreed that HPDs was uncomfortable to wear, 92% agreed that losing their hearing would make communication difficult (Table 2). Overall, participants displayed positive responses (73%) towards self-efficacy.

Furthermore, multivariate regression was performed on all subscales in relation to demographic variables, such as age, gender, education, job duration and job description. Results reveal that only education and job description had a significant level (*p* < 0.05, and *F* < 0.05) at the 95% confidence interval at the susceptibility, whereas the other demographic variables such as age, gender and job duration had non-significant levels (*p* > 0.05). The only demographic variable that has an association with believing their ears can ‘get toughened’ to noise is that of education as *p* = 0.00 and *F* = 0.00. All the other variables had no association as *p* > 0.05. Furthermore, multivariate regression on thinking it will be hard to hear warning signals if participants wore HPDs showed that the demographic variable education had a positive association as *p* < 0.05 (*p* = 0.02) and *F* < 0.05 (*F* = 0.03). All other demographic variables were non-significant as *p* > 0.05.

Overall, the study provides valuable insights into the attitudes and beliefs of participants regarding HL and the use of HPDs, along with the influence of demographic variables on these attitudes.

## Discussion

The principal findings of this study highlighted that participants were knowledgeable on NIHL, they knew when to wear HPDs and they knew how to fit and wear their HPDs. Furthermore, participants knew the damaging effects of NIHL.

Following the results of this study, 61% disagreed that it would be hard to hear warning signals while wearing HPDs, while 96% of the respondents agreed that they could protect their hearing by wearing HPDs. The use of HPDs is largely influenced by individuals’ attitudes and beliefs towards them, despite the reported hindrance of communication with peers (Kerr et al., 2017). Kerr and colleagues (2017) report that these attitudes and beliefs are largely shaped by the education employees receive on NIHL and as a result of the prevailing occupational health and safety culture within the industry. In industries where there is a strong emphasis on occupational safety and safety behaviours, employees are more likely to be reminded of safety practices, leading to a higher level of compliance in wearing HPDs. On the other hand, employees in industries with a low safety culture may exhibit lower compliance in wearing HPDs. The significance of these findings resides in emphasising how attitudes and beliefs affect how employees adopt HPDs. These studies reveal that despite the stated difficulty in communication while wearing HPDs, majority of participants still understand the significance of protecting their hearing by using these devices.

A study conducted in South Africa in a gold mine on the usage of HPDs concluded that 82% of participants responded that being exposed to noise can damage their hearing, while 89% reported that wearing HPDs is beneficial (Hansia & Dickson, 2010). Similarly, in this study, the severity of the consequences to HL subscale found that 95% agreed that they could prevent HL by wearing HPD. The results show that a sizable number of mine workers are aware of the potentially dangerous implications of excessive loud noise on their hearing health. This understanding is essential because encouraging preventative measures begins with acknowledging the hazards. The high level of agreement with the notion that wearing HPDs can prevent NIHL indicates that mine workers are open to implementing safer practices and may be more apt to abide by the use of HPDs. For the long-term health and wellbeing of mine workers, it is crucial that they are aware of and embrace HPDs. In addition, mine workers can protect themselves against NIHL and potentially prevent serious health problems later in life by adopting the usage of HPDs.

Moreover, the subscale on benefits of preventive action found that nearly all participants (98%) agreed that they knew when to wear HPDs, 83% agreed that their hearing was being hurt by exposure to loud noises at work, in addition to 98% agreeing that they planned to wear HPDs when they worked near loud noises. Comparably, a local study conducted in the mining industry (*n* = 88), on the usage of HPDs, concluded that 24% wore their HPDs all the time, 47% wore them daily in high noise environments, 70% wore them on some days, while 10% wore them on most days in high noise environments (Hansia & Dickson, 2010). However, the same study highlighted that 80% of participants said that they were never told about the benefits of wearing HPDs and can therefore relate this response to the low usage of HPDs, that is, 31% always used HPDs for the entire shift, while only 50% of the workforce were observed to have been wearing their HPDs (Hansia & Dickson, 2010). The aforementioned suggests that further research into the factors influencing some employees’ decisions to wear or not wear HPDs at particular periods is necessary in the light of this diversity in replies. In order to effectively increase HPD compliance and safeguard the hearing health of employees, it is essential to understand these elements. Moreover, results of the given studies emphasise the significance of knowing the fundamental factors that affect employees’ decisions on the use of HPDs and drawing attention towards the advantages of using these devices. Increased use of HPD offers greater protection against NIHL, which can only result from improving knowledge and putting in place practical measures.

In this study, nearly all participants (98%) agreed that exposure to loud noise could hurt their hearing. Similarly, one miner from a qualitative study conducted on platinum mines in Limpopo on the perceptions on factors contributing to NIHL said that he knew he had to take responsibility of his hearing by wearing earplugs, and if he didn’t, he would be ‘killing’ himself (Muthelo et al., 2019). Comparably, a study conducted in South Africa in a gold mine on the usage of HPDs concluded that 82% of participants responded that being exposed to noise can damage their hearing, while 89% reported that wearing HPDs are beneficial (Hansia & Dickinson, 2010). Likewise, a study carried out in Limpopo on the perceptions on factors contributing to NIHL among platinum miners identified that those mine workers knew about the sources of noise in their respective work areas (Muthelo et al., 2019). When considered collectively, these studies offer consistent proof that miners in various settings are aware of the potentially harmful implications of excessive loud exposure on their hearing health. This increased understanding can act as a springboard for fostering a strong safety culture and the regular use of HPDs to reduce the risk of NIHL. These studies highlight the significance of understanding the level of knowledge among mine workers regarding the dangers of noise exposure and the use of HPDs, in addition to emphasising the value of encouraging a safe work environment and offering workers the necessary information and tools to properly protect their hearing.

The subscale on barrier to preventative action concluded that 76% disagreed that they did not intend on wearing HPDs when they were around loud tools or equipment; 97% agreed that wearing HPDs every time they are in loud environments is important; and 91% agreed that most of their co-workers thought it was a good idea to wear HPDs in hazardous noise. Correspondingly, a study conducted by Mizan et al. (2014) that focused on NIHL in the iron and steel industry in South Africa, which involved a sample of 104 employees, found that employees in this study had knowledge about NIHL, and approximately 30% of them expressed concerns about the noise levels in their work environment. The study emphasises that employees are aware of the high noise intensity and the potential impact it can have on their hearing (Mizan et al., 2014). Furthermore, it underscores the significance of wearing HPDs to mitigate the risks associated with NIHL (Mizan et al., 2014). When considered as a whole, findings indicate that employees are knowledgeable of the dangers of exposure to high noise and have a favourable attitude towards employing the use of HPDs. They appreciate the value of continuously wearing HPDs in noisy environments and their co-workers’ support for this practice. With the aforementioned in mind, organisations should appreciate the significance of encouraging a safety-conscious culture by increasing awareness of the potential effects of noise on hearing health.

The subscale on social norms highlighted that majority of participants (95%) agreed that they wore HPDs whenever they worked around loud noises. This high percentage may be attributed to employees opting to wear their HPDs when they observed their colleagues doing the same. Comparatively, the study conducted by Tantranont and Codchanak (2017) on industrial workers to determine the predictors of HPD use confirms that when employees saw their colleagues wearing HPDs while working in noisy environments, it prompted them to wear their own HPDs (Tantranont & Codchanak, 2017). The aforementioned highlights the influence of societal norms on workers’ decisions to wear HPDs. Employees who see their co-workers using HPDs are more likely to adopt similar habits because of its normative influence on them. This realisation emphasises the significance of fostering an environment at work where the use of HPDs is normalised and supported by employees’ collective behaviour. Employers can improve workplace safety, encourage the use of HPDs and lower the risk of NIHL in their workforce by fostering such standards.

Nearly all participants in this study (98%) agreed that they knew how to fit and wear earplugs, which indicates a high level of understanding among the participants regarding the proper technique of using HPDs. However, contradictory to the given study results, a study conducted in South Africa in eight iron and steel plants found that while all the companies had a HCP in place, which made provisions for training on NIHL, nearly 40% of the participants failed to demonstrate the precise technique of inserting the HPD (Mizan et al., 2014). This suggests a discrepancy between the knowledge provided through the HCP and the actual application of the proper technique for wearing HPDs among some employees. This stresses the necessity for a more insightful content contained within HCPs that address the proper technique of wearing HPDs. Employers can increase the likelihood that employees will use HPDs appropriately and, as a result, improve overall compliance with hearing protection standards by providing clear recommendations on the proper handling and fitting of HPDs.

It was also found that employees’ level of education was the only demographic variable that was significant at the susceptibility to HL subscale, which highlights that a lower level of education was significantly associated with the employees’ susceptibility to HL. Although historically, mineworkers lacked formal education and training, this study highlighted that majority of respondents (51%) possessed a Grade 9–12 level of education, while only 16% of respondents had a Grade 5–8 level. Similarly, a study conducted by Ntlhakana et al. (2015) on the use of HPDs at a gold mine in South Africa highlighted that majority of respondents had primary and secondary levels of education, 19% and 42%, respectively. However, contrary to this study’s findings, the results concluded by Ntlhakana et al. (2015) state that the level of education was unrelated to the respondents’ use of HPDs and ultimately the development of NIHL. With the aforesaid in mind, organisations should be encouraged to strengthen their HCPs by including education on HPD use and on the development of NIHL if mines were to curb incidences of NIHL and increase HPD use. Degeest and colleagues (2018) further suggest that training provided as part of an organisation’s HCP should be commodity-specific, area-specific, in addition to being occupationally specific.

### Recommendations

The mine under study could use these results in building on their existing HCP to create a more holistic programme, which takes into account employees’ attitudes and beliefs. Further studies to determine employees’ attitudes and beliefs towards NIHL should be conducted using a mixed-methods approach. The qualitative, open-ended response approach will allow for real emotions to be captured and documented. And information yielded from these studies could further enhance the quality and comprehensiveness of the HCP devised from the results.

The BAHPHL questionnaire should be used and tested in other industries to see if the results of this study can be generalised or refuted as this tool was mainly used on the youth who were not in the formal working sector. Literature has highlighted a gap between how participants respond (elevating their compliance levels) and Researchers’ observations (which is mostly at a lower compliance level). Other studies using the BAHPHL questionnaire could combine a site survey to allow for factual insight when comparing observations with the data collected from respondents.

The coal mineworkers who participated in this study have a strong understanding of the detrimental consequences of prolonged exposure to loud noises. It is imperative for policymakers to consider the attitudes and beliefs of employees regarding noise, NIHL and compliance with wearing HPDs. By incorporating these factors, a comprehensive HCP can be developed, addressing the shortcomings identified in the study.

Moreover, the implementation of an effective HCP would lead to the resolution of existing gaps, ultimately resulting in increased compliance with wearing HPDs among employees. This, in turn, would contribute to a reduction in the prevalence of NIHL cases among coal mineworkers. Therefore, it is crucial for policy makers to prioritise the development and implementation of strategies that address employees’ attitudes and beliefs, aiming to improve compliance with wearing HPDs and ultimately minimising the occurrence of NIHL.

### Limitations

Data collection took place from a sample population from one coal mine in Mpumalanga. Therefore, results of this study cannot be generalised to other coal mines or other types of mines or sectors. Because a closed-ended questionnaire on a Likert-type scale was used, participants were not awarded the opportunity to voice their opinions on their attitudes and beliefs towards NIHL and HP. Moreover, the third item on the Likert-type scale, ‘unsure’, was regarded as a missing value and therefore leads to a loss of data using factor analysis. However, the objectives of the research were still met. And although the initial sample size of 306 was not met because of employees being reluctant as a result of production time constraints, 241 completed questionnaires were then used, which could have resulted in selection bias.

## Conclusion

Overall, the results of this study show that, even though the participants showed good knowledge of and favourable attitudes towards NIHL and HPDs, more needs to be performed to address barriers and tailor educational efforts based on educational backgrounds in order to further improve hearing conservation practices among the target population. Designing enhanced HCPs to protect employees’ hearing health and lowering the prevalence of NIHL cases in relevant businesses can benefit from the positive responses and insights acquired from this study.

## Figures and Tables

**FIGURE 1-A1:**
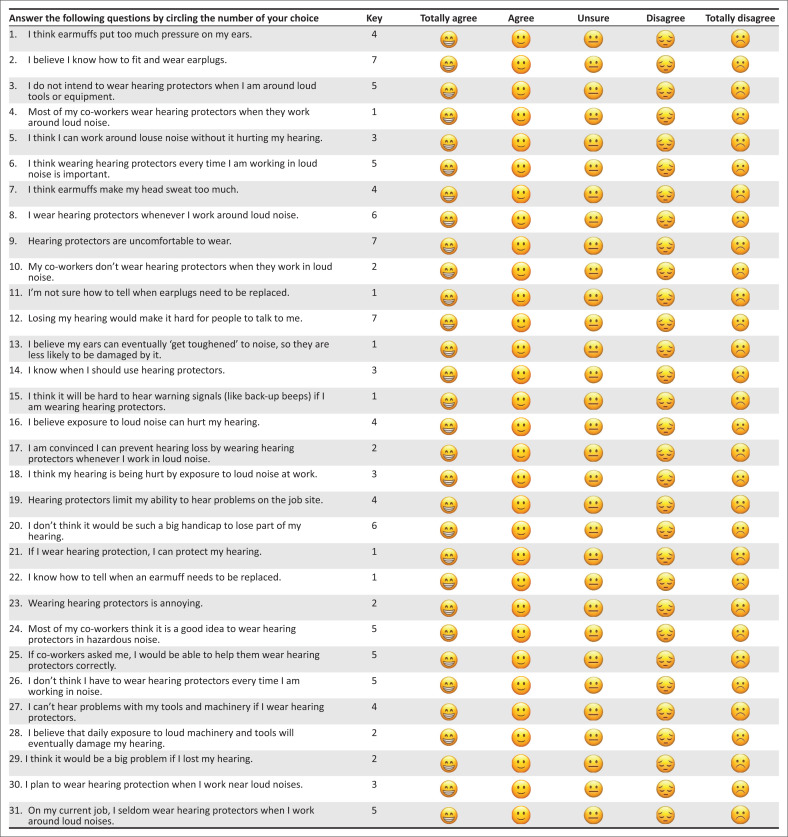
Beliefs about hearing loss and hearing protection with key.

**FIGURE 2-A1:**
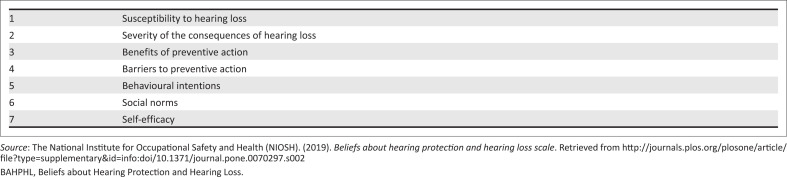
Key to BAHPHL Questionnaire.

## Appendix 1

### Responses to beliefs about hearing protection and hearing loss questionnaire per subscale

**TABLE 1-A1:** Responses to susceptibility to hearing loss.

Questions	Totally agree		Agree		Unsure		Disagree		Totally disagree	
	*n*	%	*n*	%	*n*	%	*n*	%	*n*	%
Most of my co-workers wear hearing protectors when they work around loud noise.	118	49	89	37	19	8	10	4	5	2
I’m not sure how to tell when earplugs need to be replaced.	37	15	35	15	15	7	95	39	59	24
I believe my ears can eventually ‘get toughened’ to noise, so they are less likely to be damaged by it.	47	20	48	20	17	7	57	24	72	30
I think it will be hard to hear warning signals (like back-up beeps) if I am wearing hearing protectors.	44	18	39	16	12	5	98	41	48	20
If I wear hearing protection, I can protect my hearing.	152	63	79	33	4	2	4	2	2	0
I know how to tell when an earmuff needs to be replaced.	111	46	92	38	20	8	11	15	7	3

**TABLE 2-A1:** Responses to severity of the consequences of hearing loss.

Questions	Totally agree		Agree		Unsure		Disagree		Totally disagree	
	*n*	%	*n*	%	*n*	%	*n*	%	*n*	%
My co-workers don’t wear hearing protectors when they work in loud noise.	30	12	23	10	13	5	97	40	78	32
I am convinced I can prevent hearing loss by wearing hearing protectors whenever I work in loud noise.	148	61	82	34	4	3	6	2	1	0
Wearing hearing protectors is annoying.	32	13	36	15	15	6	91	38	67	28
I believe that daily exposure to loud machinery and tools will eventually damage my hearing.	140	58	83	34	6	3	10	5	2	0
I think it would be a big problem if I lost my hearing.	160	66	70	29	6	3	2	1	3	1

**TABLE 3-A1:** Responses to benefits of preventive action.

Questions	Totally agree		Agree		Unsure		Disagree		Totally disagree	
	*n*	%	*n*	%	*n*	%	*n*	%	*n*	%
I think I can work around loud noise without hurting my hearing.	40	17	38	16	8	3	76	32	79	33
I know when I should use hearing protectors.	147	61	89	37	2	1	2	1	1	0
I think my hearing is being hurt by exposure to loud noise at work.	106	44	94	39	12	5	22	9	7	3
I plan to wear hearing protection when I work near loud noises.	159	66	78	32	1	0	0	0	3	1

**TABLE 4-A1:** Responses to barriers to preventive action.

Questions	Totally agree		Agree		Unsure		Disagree		Totally disagree	
	*n*	%	*n*	%	*n*	%	*n*	%	*n*	%
I think I can work around loud noise without hurting my hearing.	40	17	38	16	8	3	76	32	79	33
I know when I should use hearing protectors.	147	61	89	37	2	1	2	1	1	0
I think my hearing is being hurt by exposure to loud noise at work.	106	44	94	39	12	5	22	9	7	3
I plan to wear hearing protection when I work near loud noises.	159	66	78	32	1	0	0	0	3	1

**TABLE 5-A1:** Responses to behavioural intentions.

Questions	Totally agree		Agree		Unsure		Disagree		Totally disagree	
	*n*	%	*n*	%	*n*	%	*n*	%	*n*	%
I do not intend to wear hearing protectors when I am around loud tools or equipment.	31	13	24	10	4	2	94	39	88	37
I think wearing hearing protectors every time I am working in loud noise is important.	156	65	78	32	3	1	2	1	2	1
Most of my co-workers think it is a good idea to wear hearing protectors in hazardous noise.	129	54	89	37	11	5	10	4	2	1
If co-workers asked me, I would be able to help them wear hearing protectors correctly.	144	60	89	37	4	2	1	0	3	1
I don’t think I have to wear hearing protectors every time I am working in noise.	33	14	20	8	2	1	85	35	101	42
On my current job, I seldom wear hearing protectors when I work around loud noises.	81	34	43	18	5	2	50	21	62	26

**TABLE 6-A1:** Responses to social norms.

Questions	Totally agree		Agree		Unsure		Disagree		Totally disagree	
	*n*	%	*n*	%	*n*	%	*n*	%	*n*	%
I think I can work around loud noise without hurting my hearing.	40	17	38	16	8	3	76	32	79	33
I know when I should use hearing protectors.	147	61	89	37	2	1	2	1	1	0
I think my hearing is being hurt by exposure to loud noise at work.	106	44	94	39	12	5	22	9	7	3
I plan to wear hearing protection when I work near loud noises.	159	66	78	32	1	0	0	0	3	1

**TABLE 7-A1:** Responses to self-efficacy.

Questions	Totally agree		Agree		Unsure		Disagree		Totally disagree	
	*n*	%	*n*	%	*n*	%	*n*	%	*n*	%
I believe I know how to fit and wear earplugs.	137	57	99	41	2	1	0	0	3	1
Hearing protectors are uncomfortable to wear.	32	13	36	15	11	5	98	41	64	27
Losing my hearing would make it hard for people to talk to me.	140	58	81	34	8	3	8	3	4	2
